# Anticancer and antimicrobial potential of enterocin 12a from *Enterococcus faecium*

**DOI:** 10.1186/s12866-021-02086-5

**Published:** 2021-02-04

**Authors:** Preeti Sharma, Sumanpreet Kaur, Bhupinder Singh Chadha, Raminderjit Kaur, Manpreet Kaur, Sukhraj Kaur

**Affiliations:** 1 Department of Microbiology, Guru Nanak Dev University, Amritsar, Punjab 143005 India; 2 Department of Molecular Biology and Biochemistry, Guru Nanak Dev University, Amritsar, Punjab India; 3 Department of Human Genetics, Guru Nanak Dev University, Amritsar, Punjab India

**Keywords:** Bacteriocin, Enterocin, *Enterococcus faecium*, Anticancer, Commensal, Antimicrobial

## Abstract

**Background:**

Increase in the number of infections caused by Gram-negative bacteria in neutropenic cancer patients has prompted the search for novel therapeutic agents having dual anticancer and antimicrobial properties. Bacteriocins are cationic proteins of prokaryotic origin that have emerged as one of the most promising alternative antimicrobial agents with applications as food preservatives and therapeutic agents. Apart from their antimicrobial activities, bacteriocins are also being explored for their anticancer potential.

**Results:**

In this study, a broad-spectrum, cell membrane-permeabilizing enterocin with a molecular weight of 65 kDa was purified and characterized from the culture supernatant of vaginal *Enterococcus faecium* 12a. Enterocin 12a inhibited multidrug-resistant strains of various Gram-negative pathogens such as *Salmonella enterica*, *Shigella flexneri*, *Vibrio cholerae*, *Escherichia coli* and Gram-positive, *Listeria monocytogenes,* but had no activities against different strains of gut lactobacilli. The mass spectrometric analysis showed that the enterocin 12a shared partial homology with 4Fe-4S domain-containing redox protein of *E. faecalis* R712. Further, enterocin 12a selectively inhibited the proliferation of various human cancer cell lines in a dose-dependent manner but not that of normal human peripheral blood mononuclear cells. Enterocin 12a-treated cancer cells showed apoptosis-like morphological changes.

**Conclusion:**

Enterocin 12a is a novel bacteriocin that has anticancer properties against human cell lines and negligible activity towards non-malignant cells. Therefore, it should be further evaluated for its anticancer potential in animal models.

**Supplementary Information:**

The online version contains supplementary material available at 10.1186/s12866-021-02086-5.

## Background

Cancer is the second most prevalent cause of death worldwide that accounted for 9.6 million deaths, and 18.1 million new cases in 2018 [1]. Conventional cancer chemotherapeutic agents are mostly non-selective to cancer cells that result in serious side-effects such as organ toxicity, immunosuppression, and also contribute to the development of drug resistance in cancer cells. Immunosuppression due to anticancer drug regimen and breakdown in the mucosal barrier due to the use of invasive devices in cancer patients make them susceptible to bacterial infections that require long-term prophylactic antibiotic regimens [2]. Further, the effectiveness of antibiotic regimen to treat infections in cancer patients is compromised due to the emergence of drug-resistant Gram-negative bacterial pathogens that predominates in neutropenic patients [3]. Therefore, novel anticancer agents that specifically inhibit cancer cells and have dual anticancer and antimicrobial activities are expected to act as prophylaxis against bacterial infections along with inhibiting the growth of tumors in cancer patients [4].

Exploration for novel antimicrobials to treat bacterial infections has led to renewed interest in the search for bacteriocins [5]. Bacteriocins are cationic proteins secreted by bacteria that kill the target bacteria mainly by forming pores in their cell membrane or in some cases by inhibiting protein synthesis and DNA replication [6]. Bacteriocins derived from lactic acid bacteria (LAB) have GRAS (Generally regarded as safe) status and therefore they are considered safe. Two such bacteriocins, nisin and pediocin have been approved by the Food and Drug Administration (FDA) for use as food preservatives [7]. Bacteriocins are also being considered as potential new generation therapeutic agents that can be employed as stand-alone drugs [6] or as adjuncts to conventional antibiotics [8, 9]. In healthcare studies, nisin was shown to successfully treat mastitis in humans [10] and cows [11].

Along with the antimicrobial activities, a few bacteriocins of LAB origin such as nisin [12], pediocin, and plantaricin have demonstrated anti-proliferative effects against human cancer cell lines, in in vitro and mice experiments [13]. This indicates their potential for use as both antimicrobial and anticancer agents. However, bacteriocins purified from LAB generally have narrow-spectrum antimicrobial activities as they are known to inhibit Gram-positive bacteria only [5, 14]. Therefore, bacteriocins that inhibit Gram-negative pathogens along with Gram-positive are expected to have wider applications. Thus, this study was focused on studying the anticancer potential of a broad-spectrum enterocin purified from the culture supernatant (CS) of *E. faecium* 12a. Further, the mode of anticancer activity and toxicity of the enterocin was also studied.

## Results

### Cumulative production kinetics of the antimicrobial substance in the CS

**Fig. 1:**
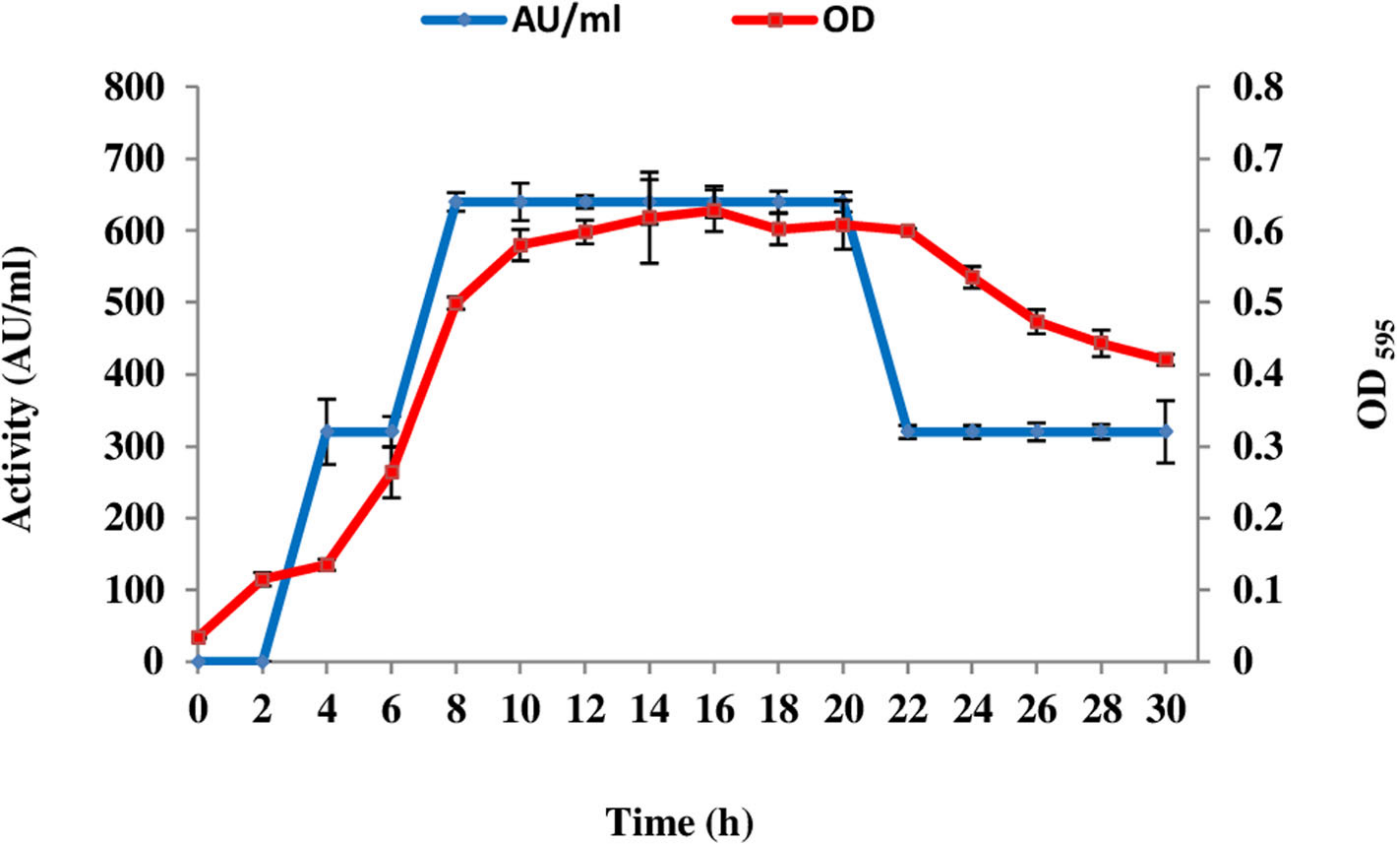
Kinetics of enterocin production and growth kinetics of *E. faecium* 12a.  The antimicrobial activity of CS was measured by using agar gel diffusion method against *S. enterica*, whereas, the growth of *E. faecium* in MRS media was measured by measuring absorbance at 595 nm after every 2 h. Error bars are representative of the three independent experiments performed in triplicates

Before the purification of enterocin 12a, its production kinetics was studied in De Man Rogosa and Sharpe medium (MRS) broth. As shown in Fig. 1, enterocin 12a appeared in the CS 4 h after the inoculation of *E. faecium* 12a in MRS broth. The concentration of 12a peaked (640 arbitrary units per ml, AU/ml) at 8 h after which it plateaued till 20 h. After 20 h, there was a 50% decrease in the antimicrobial activity of the CS. The production of the antimicrobial substance was growth-associated as it peaked in the early log phase and plateaued in the stationary phase.

### Purification and identification of enterocin 12a

**Fig. 2:**
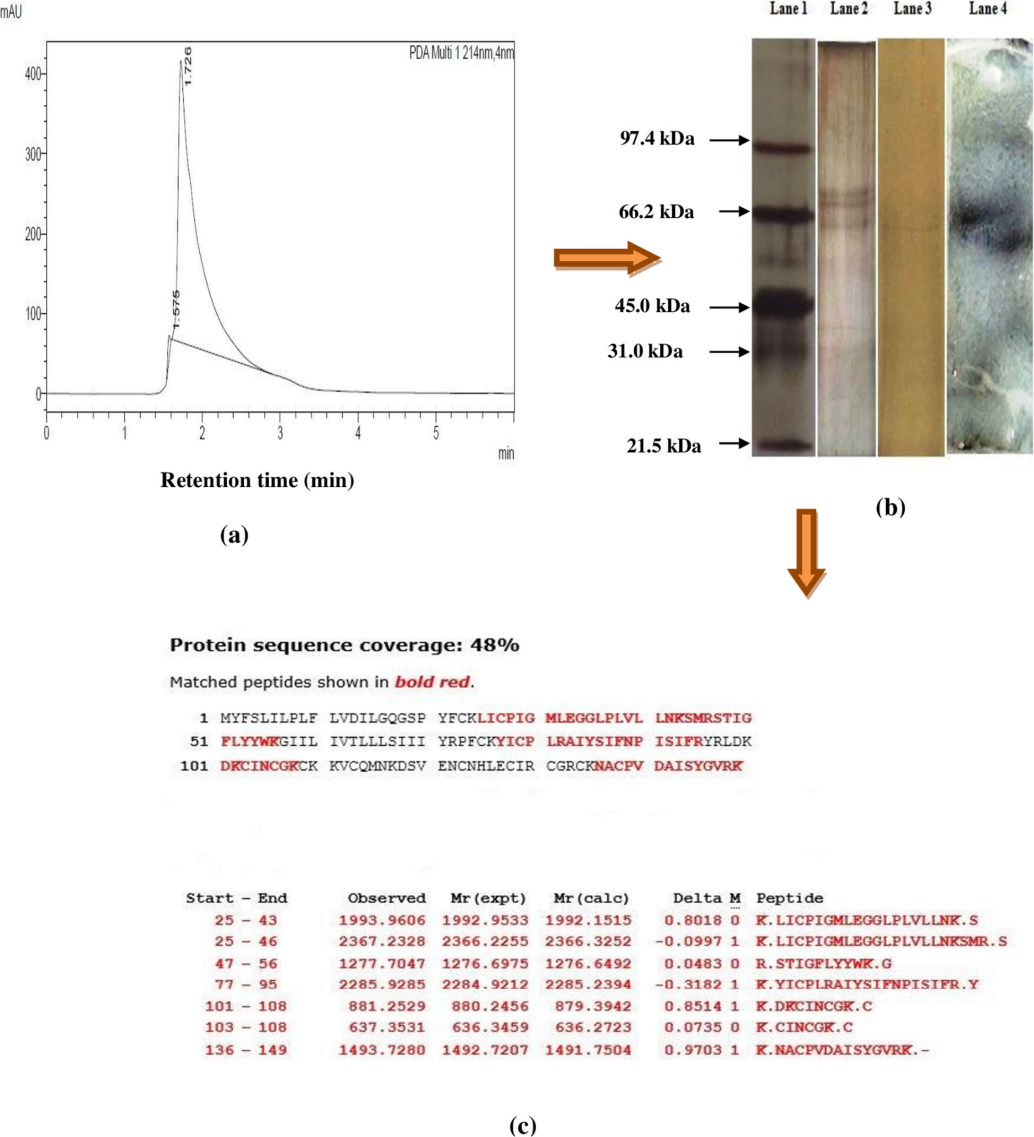
**(a)** U-HPLC chromatogram of the purified enterocin 12a **(b)** SDS-PAGE of purified enterocin 12a and gel overlay with *S. enterica*. Lane 1: molecular weight marker. Lane 2: partially-purified enterocin 12a after cation exchange chromatography. Lane 3: purified enterocin 12a after RP-HPLC. Lane 4: Zone of inhibition against *S. enterica* observed in the agar gel overlay assay corresponding to the position of the band on the SDS-PAGE of enterocin 12a **(c)** Peptide mass fingerprinting (PMF) analysis of enterocin 12a. The PMF followed by MASCOT search in NCBIprot shows the matched amino acid residues (in red bold) of the seven peptide fragments with the 4Fe-4S domain-containing protein of *E. faecalis* R712

For the purification of antimicrobial compound from the CS, ammonium sulfate precipitation, cation-exchange chromatography and reverse-phase high-performance liquid chromatography (RP-HPLC) was carried out. Purified enterocin fraction yielded a single peak on the chromatogram at the retention time of 1.72 min (Fig. 2a). After RP-HPLC, a 10.11 fold increase in the specific activity of enterocin was observed and the final yield percentage was 1.6 (Table 1). SDS-PAGE analysis of the purified fraction showed a single band with a molecular weight of 65 kDa (Fig. 2b; Supplementary Fig. 1, Supplementary Fig. 2). Further, to test the antimicrobial activity of the 65 kDa band, gel overlay assay was performed against *Salmonella enterica* as the indicator pathogen, and a clear zone corresponding to the band was obtained (Fig. 2b; Supplementary Fig. 3). The band was cut and subjected to MALDI TOF/TOF MS/MS analysis. Protein identification by MASCOT search in NCBIprot showed that the peptide fragments of enterocin 12a shared similarity with 48% of 4Fe-4S domain-containing protein of *E. faecalis* R712 with significant (*p*< 0.05) coverage score of 121(Fig. 2c). The seven peptides that matched the 4Fe-4S domain-containing protein of *E. faecalis* R712 are listed in Fig. 2c.

**Table 1:** Steps of purification and percent yield of enterocin 12a purified from *E. faecium* 12a

Purification step	Total volume (ml)	Activity (AU/ml)	Total activity^**a**^	Total Protein (mg)	Specific activity^**b**^	Fold-Increase in the specific activity	Percent Yield
**CS**	1000	68,266	6.8× 10^7^	625	108,800	1	100
**Ammonium sulphate ppt.**	200	136,528	2.7×10^7^	215	125,581	1.15	39.7
**Cation-exchange fraction**	30	273,056	8.1× 10^6^	33	245,455	2.26	11.9
**Reverse-phase chromatography**	2	546,112	1.1×10^6^	1	1,100,000	10.11	1.6

^a^Total activity= Activity × Total volume of sample

^b^Specific activity = Total activity/ Total protein

The antimicrobial activities of both CS and purified enterocin 12a were determined against various pathogens and commensal lactobacilli strains. Results showed that both crude and purified enterocin 12a inhibited Gram-negative pathogenic strains such as *S. enterica*, *Shigella flexneri*, *Escherichia coli*, and *Vibrio cholerae* but did not inhibit Gram-positive bacteria such as *Staphylococcus aureus*, *Streptococcus pyogenes,* and lactobacilli spp., except *Listeria monocytogenes*. Further, CS also inhibited *Mycobacterium smegmatis* but purified enterocin 12a had no activity against *M. smegmatis* (Table 2).

**Table 2:** Antimicrobial activity of the CS and enterocin 12a against various indicator bacterial strains

Indicator bacteria	Zone of inhibition (mm)	
	CS	Enterocin 12a
*S. enterica* MTCC 733	16±0.1	15±0.1
*Esc. coli* MTCC 119	13±0.2	13±0.2
*Sh. flexneri* MTCC 1457	15±0.2	15±0.2
*List. monocytogenes* MTCC 657	14±0.3	13±0.3
*V. cholerae* MTCC3906	13±0.3	12±0.3
*M. smegmatis* MTCC6	14±0.2	-*
*St. pyogenes* MTCC 1927	–	–
*Staph. aureus* MTCC 96	–	–
*Pseudomonas aeruginosa* MTCC 741	–	–
*Lactobacillus plantarum* L12	–	–
*L. plantarum* L14		
*L. paracasei* L32	–	–
*L. pentosus* S45	–	–
*L. fermentum* L13	–	–
*L*. *fermentum* L18	–	–
*L*. *casei* S49	–	–

*-No zone of inhibition observed

The zone of inhibition (mm) of CS and enterocin 12a against indicator bacterial strains was determined by using agar gel diffusion assay. The results are the mean±standard deviation (SD) of three independent experiments performed in triplicates.

### Physico-chemical characteristics of purified enterocin 12a

The thermostability of purified enterocin 12a was determined at different temperatures. As shown in Table 3, the antimicrobial activity of enterocin 12a remained stable at temperatures 60 and 80 °C; however, the enterocin was completely inactivated beyond 30 min exposure to a temperature of 100 °C. Further, the pH sensitivity of enterocin 12a was determined. Results showed that enterocin 12a remained active in the pH range 2–10 and exhibited maximum activity at pH 4 (Table 3). Further, the antimicrobial activity of enterocin 12a was stable to the action of various solvents such as methanol, chloroform, and acetonitrile (Table 3).

**Table 3:** Physico-chemical characterization of enterocin 12a

Physico-chemical parameters	Treatments	Residual activity of enterocin 12a in terms of zone of inhibition (mm)
	Untreated control	15±0.11
**Temperature**	60 °C (30 min)	15±0.12
	60 °C (60 min)	15±0.20
	80 °C (30 min)	14±0.20
	80 °C (60 min)	13±0.11
	100 °C (15 min)	8±0.15
	100 °C (30 min)	*-
	121 °C (15 min)	–
**pH**	2	13±0.14
	4	14±0.12
	6	13±0.15
	8	12±0.13
	10	11±0.12
**Solvents (50%v/v)**	Chloroform	14±0.11
	Methanol	15±0.11
	Acetonitrile	15±0.11
**Enzymes (1 mg/ml)**	Proteinase K	–
	Pepsin	–
	Trypsin	–
	Lipase	15±0.12

*-No zone of inhibition observed

The effect of various enzymes on the stability of enterocin 12a was tested. It was observed that all three proteases resulted in complete abrogation of the antimicrobial activity of enterocin 12a, thereby showing its proteinaceous nature (Table 3). On the other hand, lipase treatment did not affect the antimicrobial activity of enterocin 12a (Table 3).

Zones of inhibitions (mm) of enterocin 12a were measured by using agar gel diffusion assay against *S. enterica*. The results are the mean±SD of three independent experiments performed in triplicates.

### Effect of enterocin 12a on membrane permeability

**Fig. 3:**
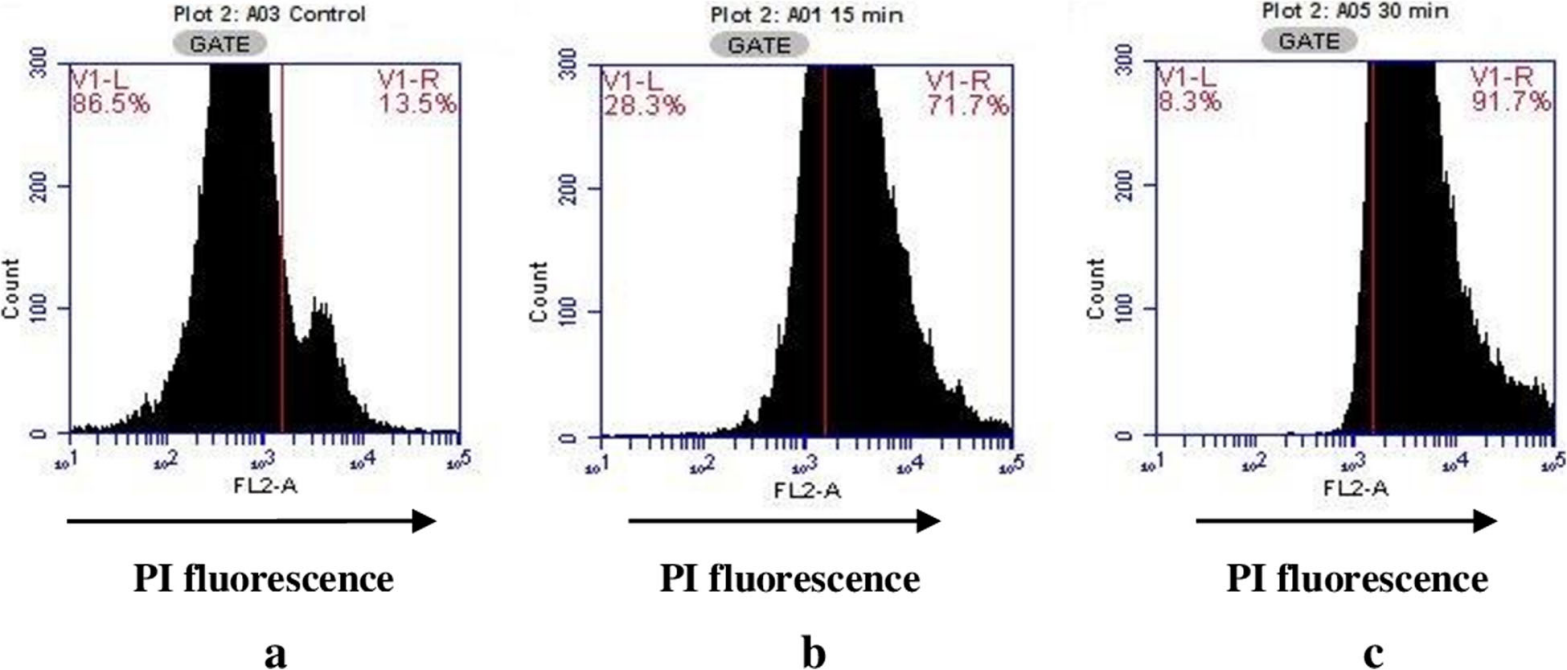
Histograms depicting PI fluorescence of *S. enterica* cells **a)** untreated control **b)** enterocin 12a-treatment for 15 min **c)** enterocin 12a-treatment for 30 min. The cells of *S. enterica* were treated with 5 μg/ml of enterocin for 15 and 30 min. The cells were washed by centrifugation and stained with PI. The fluorescence of the PI-stained cells was determined by flow cytometer. Data are represented as histograms with counted bacterial events displayed on y-axis, and increase in fluorescence on the x-axis

**Fig. 4:**
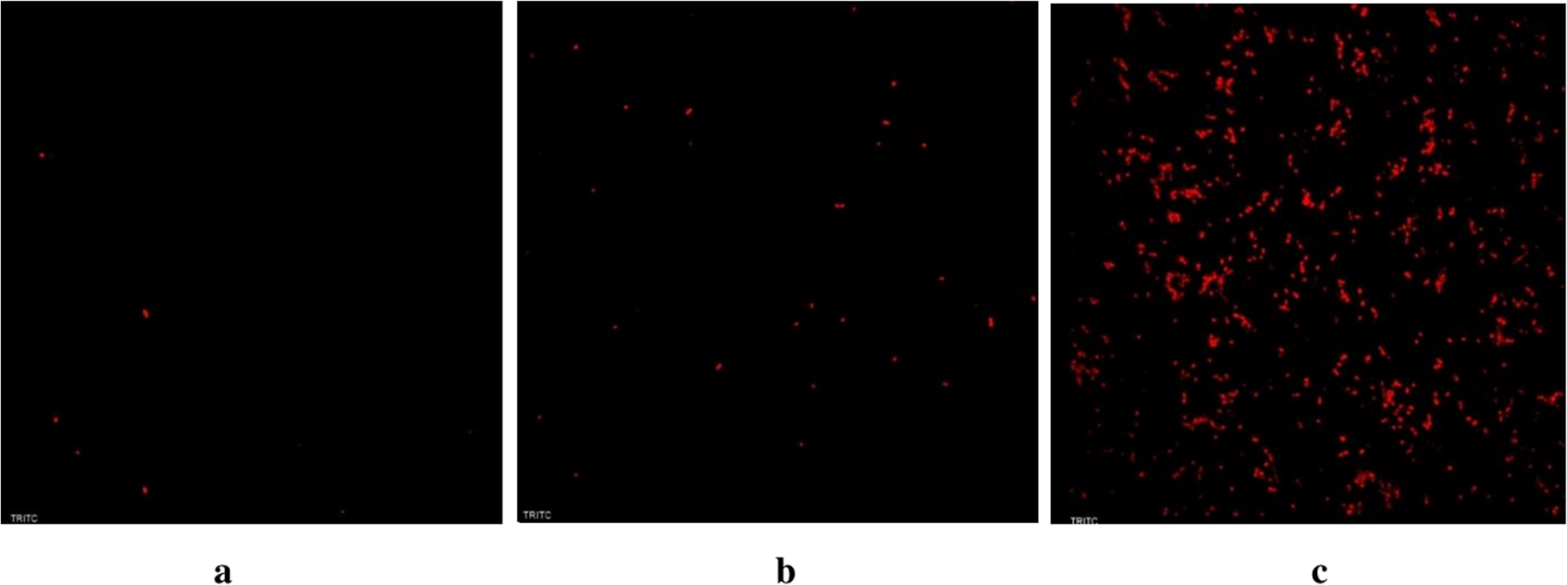
Confocal microscopy (magnification 1000 X) images of PI-stained *S. enterica* cells **(a)** without enterocin 12a treatment **(b)** with enterocin 12a (5 μg/ml) treatment for 15 min **(c)** with enterocin 12a (5 μg/ml) treatment for 30 min

The effect of enterocin 12a on cell membrane permeability of *S. enterica* cells was determined by staining the cells with the fluorescent dye, propidium iodide (PI). Histograms (Fig. 3a) show that in the absence of enterocin 12a, *S. enterica* cells exhibited 13.5% fluorescence. Whereas, in the presence of enterocin 12a, the fluorescence of *S. enterica* cells increased to 71.7 and 91.7% after 15 and 30 min, respectively (Fig. 3b and c). Simultaneously, the confocal microscopy images (Fig. 4) of enterocin 12a-treated *S. enterica* cells stained with PI showed an exponential increase in the number of fluorescent cells with an increase in the treatment time as compared to the untreated cells (Fig. 4).

### Hemolytic activity of enterocin 12a

**Fig. 5:**
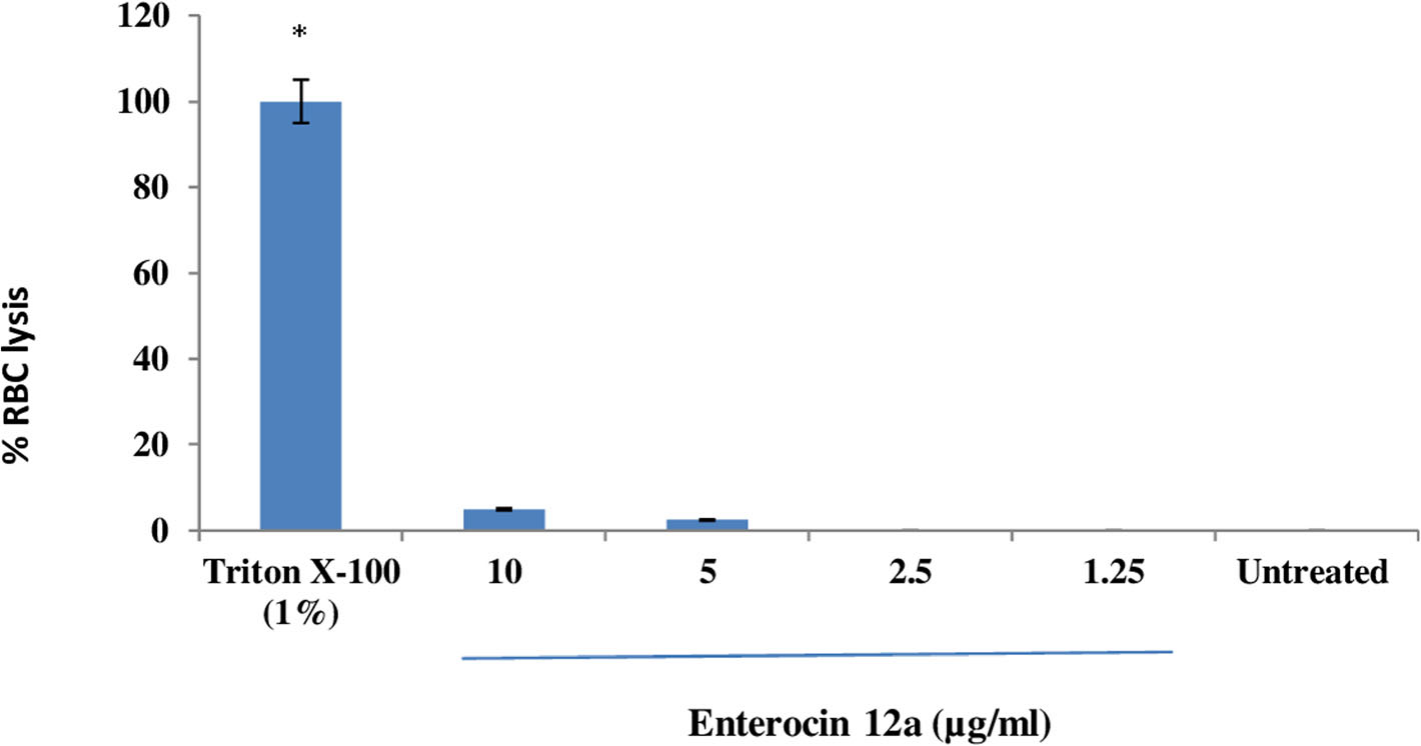
Hemolytic activity of enterocin 12a. Red blood cell samples suspended in PBS were treated with different concentrations of enterocin 12a for 1 h. Triton X-100 (1%) was used as a positive control. Error bars are representative of SD of the three independent experiments performed in triplicates. *Significance (*P* < 0.0001) was calculated by comparing all the groups to the untreated control by using independent Student’s *t*-test

The toxicity of enterocin 12a was determined by testing its hemolytic activity against human red blood cells (RBCs). Results (Fig. 5) showed that the treatment of RBCs with 2-fold dilutions of enterocin did not result in hemolysis. The percent RBC lysis observed after treatment with 10 and 5 μg/ml of enterocin was 4.5 and 2.5%, respectively which was not significant (*p* < 0.0001) as compared to the untreated RBCs. On the other hand, treatment with 1% Triton X-100 resulted in complete RBC lysis (Fig. 5).

### Anti-proliferative activity of enterocin 12a

**Fig. 6:**
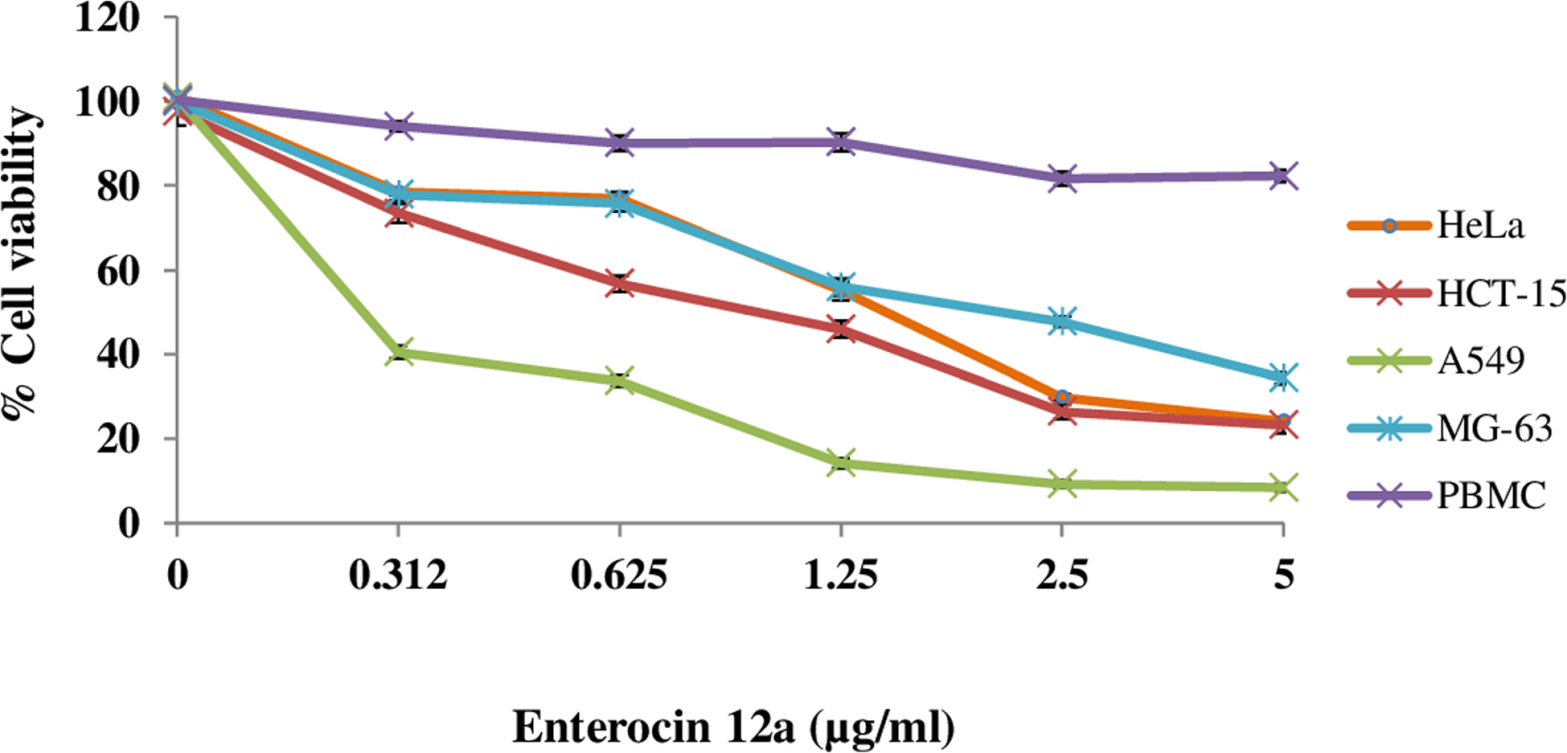
The anti-proliferative effects of different concentrations of enterocin 12a on the viabilities of cell lines A549, HeLa, HCT-15, MG-63 and PBMC as demonstrated by MTT assay. Error bars are representative of SD of the three independent experiments. *Significance (P < 0.0001) was calculated by comparing all the groups to the untreated control by using independent Student’s *t*-test

Evaluation of the antiproliferative activities of purified enterocin 12a on the cancer cell lines such as cervical cancer cell line, HeLa; colonic epithelial cell line, HCT-15; lung cancer cell line, A549 and osteosarcoma cell line, MG-63 was done using MTT (3–4, 5-dimethylthiazol-2-yl)-2, 5-diphenyltetrazolium) assay. Enterocin 12a inhibited the growth of all the cell lines in a dose-dependent manner (Fig. 6). At the highest dose of 5 μg/ml, enterocin 12a reduced the viabilities of A549, HeLa, HCT-15, and MG-63 to 8.4, 24.1, 23.2 and 34.3% respectively (Fig. 6). The 50% inhibitory concentration (IC_50_) values of the enterocin 12a for A549, HeLa, HCT-15, and MG-63 cell lines was calculated as 0.08, 1.54, 1.07 and 2.1 μg/ml, respectively. The antiproliferative effect of enterocin 12a against normal human peripheral blood mononuclear cells (PBMCs) was also determined. The residual viability of PBMCs after treatment with enterocin 12a was 82.2% that was not significant (*p*< 0.05) as compared to the untreated control (Fig. 6).

### Microscopic detection of morphological changes in the CS-treated cell lines

**Fig. 7:**
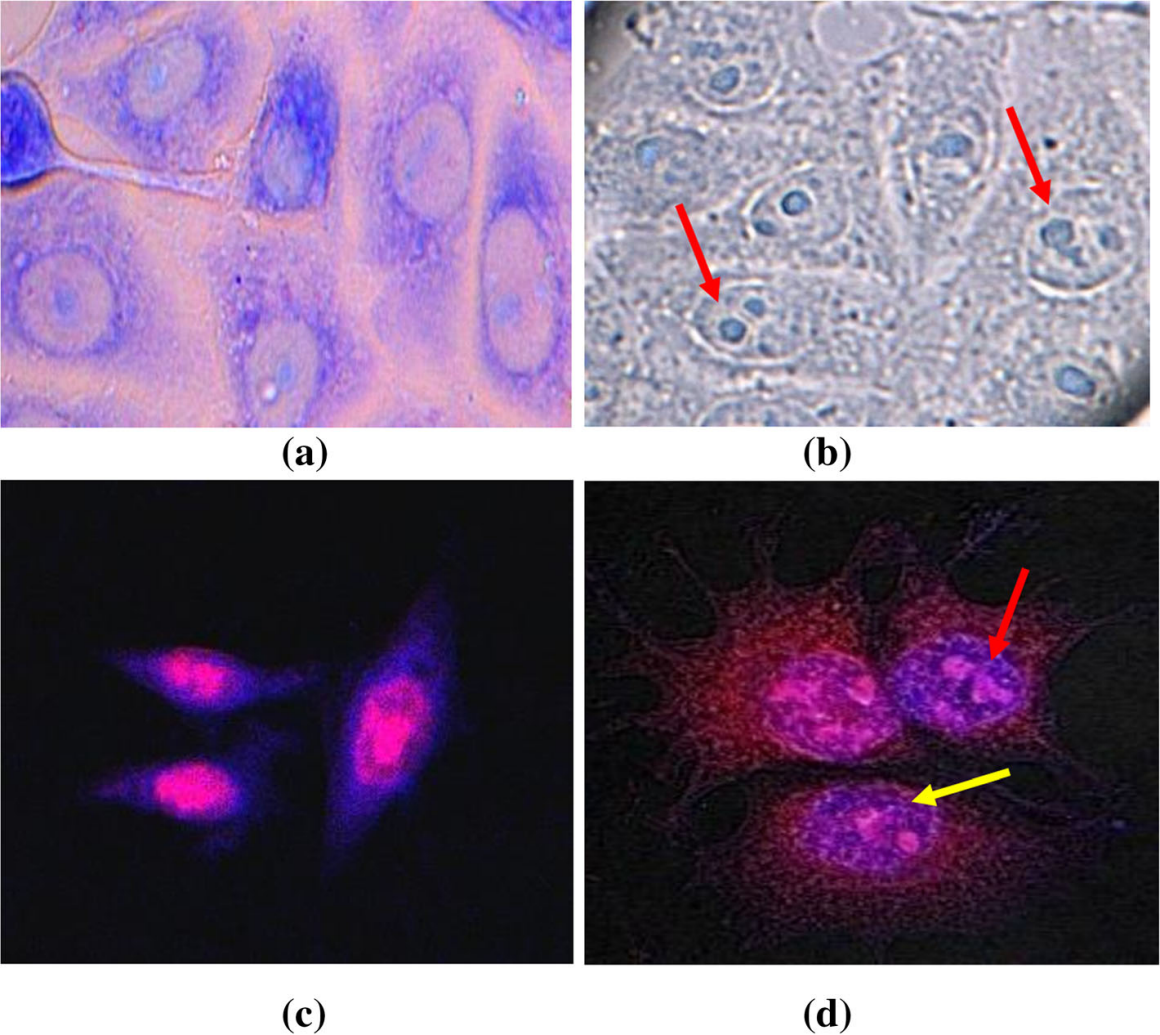
Morphological changes induced in HCT-15 after 24 h treatment with enterocin 12a (1 μg/ml). (A) Giemsa-stained untreated control cells (B) Giemsa-stained enterocin-treated cells (C) PI and Hoechst 33342 co-stained untreated control cells (D) PI and Hoechst 33342 co-stained enterocin-treated cells. Yellow arrows show cell shrinkage, and red arrows show nuclear fragmentation

Further, to study the mode of antiproliferative activity of enterocin 12a, morphological changes of enterocin 12a-treated HCT-15 cells were studied by bright field and fluorescent microscopy. Most of the cells in the control untreated sample remained viable with normal cell morphology having a clear outline of the cell membrane and nucleus (Fig. 7a and c). On the other hand, cells treated with enterocin 12a showed morphological changes typical of apoptosis (Fig. 7b and d). Enterocin 12a-treated and Giemsa-stained cells (Fig. 7b) showed chromatin condensation and nuclear fragmentation. Similarly, cells stained with fluorescent dyes, PI and Hoechst 33342 (7D) showed cell shrinkage and nuclear fragmentation.

## Discussion

Eighty percent of the patients with hematologic malignancies and 10–15% of the patients with solid tumors develop infections associated with neutropenia following more than one chemotherapy cycle [15]. Many studies have shown the benefits of using antimicrobials in conjunction with anticancer agents in reducing the mortality rate in cancer patients [3]. Thus, in this study, a novel enterocin 12a having selective anticancer activities against malignant cells was purified and characterized. *E. faecium* 12a was selected for the purification of enterocin as it possessed broad-spectrum antimicrobial activity, good probiotic properties, and did not harbor genes for known virulence factors as shown in our previous study [16]. Enterocin 12a was characterized as a novel high molecular weight protein that inhibited several Gram-negative pathogens but had no activities against Gram-positive bacteria such as *St. pyogenes*, *Staph. aureus* and commensal lactobacilli, except *L. monocytogenes*. Among the already known enterocins of *E. faecium, *enterocin E-760 [17]*,* T1 [18], enterocin A and B [19] were known to exhibit antimicrobial activities against Gram-negative bacteria; however, all of them had molecular weights of less than 10 kDa. Secondly, their antimicrobial spectrum differed from enterocin 12a, as they all inhibited *Staph. aureus*. Enterocins A and B also inhibited several strains of lactobacilli. Other enterocins of *E. faecium* such as AS48 and enterocin P [20] inhibited Gram-positive bacteria only. AS48 was shown to inhibit Gram-negative bacteria only in combination with treatments that disrupted the outer membrane of Gram-negative bacteria such as the use of EDTA, tripolyphosphate, polymyxin B, or pulsed electric fields [21].

Further, both the CS and the purified enterocin inhibited *S*. *enterica*, *Sh*. *flexneri, Esc. coli,* and *V. cholerae*, all of which cause serious food or waterborne gut infections such as enteric fever and diarrhea [22]. However, in the case of *M. smegmatis*, the CS but not the purified enterocin exhibited the antimicrobial activity. This indicated that non-proteinaceous substances present in the CS might be responsible for the antimicrobial activity against *M. smegmatis*. The antibiotic sensitivity profiles of the pathogenic indicator strains such as *S*. *enterica* (Supplementary Table 1), *Esc. Coli* (Supplementary Table 2), *Sh*. *flexneri* (Supplementary Table 3) and *V. cholerae* (Supplementary Table 4) used in the study revealed that all the four isolates were multi-drug resistant (MDR) pathogens as they exhibited resistance to at least one antibiotic in more than one antibiotic class [23]. Thus, considering the paucity of new antimicrobials against the MDR strains of Gram-negative pathogens [24], both *E. faecium* 12a and its enterocin should be further tested for their clinical efficacies in vivo infection models.

The kinetics of cumulative production of enterocin produced by *E. faecium* 12a in the MRS media was studied. Like other bacteriocins, enterocin 12a followed primary metabolite production kinetics [25], as it appeared in the CS in the early logarithmic phase and peaked in the late-log phase. Its concentration in the CS remained stable till 20 h, after which it decreased by 2-fold. The decrease in the antimicrobial activity of CS may be due to the expression of transmembrane immunity proteins that inactivate enterocin and protects the producer cells from autolysis as observed in the case of enterocin P [20] and cytolysin [26].

The physicochemical characterization of enterocin 12a showed that it completely lost its antimicrobial activity on treatment with all the 3 proteolytic enzymes, thereby showing its proteinaceous nature. Further, the exposure of enterocin 12a to the temperature of 100 °C for more than 15 min resulted in complete abrogation of its antimicrobial activity. As most of the heat-stable enterocins are resistant to temperatures as high as 121 °C, enterocin 12a can be considered heat-labile. Owing to its heat-labile nature and high molecular weight, enterocin 12a may be classified as class IV enterocin [27]. Enterocin 12a was also found to be resistant to pH ranging between 2 and 10, and all the tested organic solvents.

High molecular weight enterocins were earlier reported from various strains of *E. faecalis* viz., enterolysin A 34 kDa [28];, bacteriocin 41 64.5 kDa [29];, EF478 45 kDa [30]; and anti-*Candida* protein 43 kDa [31];. However, in the case of *E. faecium*, a single report showed the production of two high molecular weight bacteriocins (55 and 65 kDa) from the strain *E. faecium* ICIS_7_. The enterocins of *E. faecium* ICIS_7_ were shown to bear no similarities with the previously reported proteins in the protein databases [32]. In this study, the characterization of enterocin 12a by using mass spectrometry revealed that it shared homology with 48% of 4Fe-4S domain-containing protein of *E. faecalis* R712. Bacterial ferredoxin i.e., 4Fe-4S cluster-containing proteins are mostly acidic proteins that possess a high negative redox potential. They use iron-sulfur clusters as electron distributors in various metabolic pathways [33]. Another study reported the purification of ferredoxin-domain-containing bacteriocins, pectocins M1 and M2 from *Pectobacterium* spp. [34]. Pectocins were shown to be natural modular proteins containing a cytotoxic domain fused to a ferredoxin-like protein that binds to the ferrichrome transporter and causes pore formation in the target cell membrane [35]. Similar natural modular bacteriocins that bind to ferrichrome transporter and cause cytolysis of target strains were also reported from *Pseudomonas* spp. [36]. Thus, enterocin 12a might resemble such natural modular antimicrobial proteins reported only in Gram-negative bacteria till date. *E. faecium* in particular are known to possess a high rate of gene recombination [37] that might explain the presence of such natural modular enterocin in *E. faecium* 12a. Furthermore, enterocin 12a appeared to be a membrane-bound protein as it was released in the CS only in the presence of Tween 80-containing MRS media or after low-frequency ultrasonication (120 kHz) of *E. faecium* cells cultured in MRS media without Tween 80 (data not shown). A previous study has also shown that the use of Tween 80 in the broth media resulted in an 8-fold increase in the concentration of lacticin RM in the CS [38].

Further, the mode of the antimicrobial activity of enterocin 12a was studied by flow cytometry. Results showed that the treatment of *S. enterica* cells with the purified enterocin rapidly killed the cells by altering their cell membrane permeability as evidenced by 71.7 and 91.7% increase in PI fluorescence within 15 and 30 min, respectively. These results are similar to those reported for other bacteriocins such as nisin [39] and lacticin F [40].

Enterocin 12a inhibited the proliferation of all the tested cancer cell lines in a dose-dependent manner. Another enterocin i.e., enterocin A was earlier shown to reduce the viabilities of human cancer cell lines viz., HeLa, HT-29, AGS, and Caco-2 selectively by 60% at the maximum dose of 120 μg/ml [41]. However, as compared to enterocin-A, 12a appeared to be more potent as it reduced the viabilities of all the tested cell lines in the range 64–90% at a maximum dose of 5 μg/ml. Other purified bacteriocins such as pediocin PA-1 [42], plantaricin A [43], nisin ZP [44], and nisin [45] purified from LAB genera were also shown to inhibit the in vitro proliferation of human cancer cells. Nisin was shown to selectively inhibit cancer cell lines and not the normal cells [42]. Another redox bacteriocin, azurin, secreted by *P. aeruginosa* was shown to have antiproliferative effects against murine [46] and human cancer cell lines [47]. A non-toxic fragment of azurin, p28 was approved by the FDA for the treatment of malignant brain tumor, glioma [48].

Enterocin 12a was found to have negligible antiproliferative effects against human PBMCs and membranolytic effects on RBCs at the same tested doses as that of cancer cells. The selective anti-proliferative activity could be explained based on the differences between the cell membrane of cancer cells from that of normal cells. As compared to the normal cells, cancer cells are known to have increased cell membrane negative charge [49, 50], higher membrane fluidity [51], and the number of microvilli [52] which may result in enhanced binding of bacteriocins to the cell surface of the cancer cells. An attempt to study the mechanism of anticancer activity of enterocin 12a by using confocal microscopy revealed that the treatment of all the tested cancer cell lines with enterocin 12a resulted in apoptosis-like morphological changes as evident by cell shrinkage, chromatin condensation, and nuclear fragmentation [53]. Similar morphological effects on cancer cells were reported in the case of other bacteriocins such as nisin ZP [44] and azurin [54]. Cancer cells are known to possess increased amounts of negatively charged phospholipids [49, 50] on their outer cell membrane leaflet. Therefore, the anticancer effects of the enterocin 12a might be due to its cell membrane-permeabilizing effect similar to other bacteriocins [43]. However, the exact molecular mechanism of anticancer activity is under investigation.

In conclusion, these results suggest that human vaginal commensal *E. faecium* 12a secreted a novel enterocin 12a that inhibited the MDR strains of Gram-negative pathogens and also selectively inhibited the proliferation of cancer cell lines. Bacteriocin-secreting enterococci were shown to have a survival advantage in the gut, for example, bacteriocin 21-producing *E. faecalis* strain was able to persist for a longer time in the gut of mice by successfully replacing the indigenous enterococci including vancomycin-resistant strains, as compared to the strain that lacked the bacteriocin gene [55]. Thus, the therapeutic potential of both *E. faecium* 12a and its enterocin for the treatment of MDR strains of Gram-negative pathogens needs further investigation. Further, the study highlighted the functional property of a human vaginal commensal *E. faecium* that might play a role in protection against cervical cancer. Thus, the role of *E. faecium* 12a and its enterocin in protection against cancer in various in vivo models should also be explored.

## Methods

### Bacterial strains

*E. faecium* 12a used for the purification of enterocin 12a was isolated from the vaginal swab sample of the healthy pre-menopausal woman and characterized in the previous study [15]. *Lactobacillus* spp. of fecal origin used in the study was gifted by one of the authors (Sukhraj Kaur). The LAB strains were cultured in MRS media and incubated at 37 °C in anaerobic jars for growth. The bacterial growth media and the chemicals used in the study were mostly (except where mentioned), purchased from HiMedia Laboratories Pvt. Ltd., Mumbai, India.

The pathogenic bacterial strains used in this study were procured from Microbial Type Culture Collection (MTCC), Institute of Microbial Technology, Chandigarh, India. The indicator pathogenic bacteria such as *Esc. coli* MTCC 119, *P. aeruginosa* MTCC 741, *Sh. flexneri* MTCC 1457, *V. cholerae* MTCC 3906, *S. enterica Typhimurium* MTCC 733, *Lis. monocytogenes* MTCC 657, *St. pyogenes* MTCC 1927 and *Staph. aureus* subsp. *aureus* MTCC 96 were cultured in Brain Heart Infusion (BHI) broth at 37 °C under aerobic stationary conditions. Whereas, *M. smegmatis* MTCC 6 was cultured in Middlebrook 7H9 broth supplemented with bovine serum albumin fraction V and Tween 80 under aerobic conditions at 37 °C.

### Agar gel diffusion assay

The antimicrobial activities of CS and purified enterocin 12a were determined against various pathogenic and commensal lactobacilli strains by agar gel diffusion assay [56]. To prepare the CS, the overnight grown culture of *E. faecium* 12a was centrifuged (Benchtop Refrigerated centrifuge; Sigma 3 K30), at 9000×g for 10 min at 4 °C. The supernatant obtained was filter-sterilized by passing through a 0.22 μm filter, and the antimicrobial activity of the CS was determined after adjusting its pH to 6.5.

### Growth curve and kinetics of production of antimicrobial substance

To study the growth curve and kinetics of bacteriocin production by *E. faecium* 12a, MRS media was inoculated with 2% overnight grown culture of *E. faecium* and incubated at 37 °C in anaerobic jars. The growth of the culture was measured by reading its absorbance at 595 nm after every 2 h. Simultaneously, the antimicrobial activity of the CS was determined by agar gel diffusion assay against the indicator bacteria *S. enteric*a MTCC 733. The antimicrobial activity of CS was expressed in AU/ml, which is defined as the reciprocal of the highest dilution showing inhibition of the indicator lawn.

### Purification of enterocin 12a

Enterocin 12a was purified from the ammonium sulfate precipitates of the CS of *E. faecium* 12a by cation-exchange and RP-HPLC methods. For ammonium sulphate precipitation, 1 l of MRS broth was inoculated with overnight grown culture (2%) of *E. faecium* 12a and incubated at 37 °C for 16 h in anaerobic jars. The culture was centrifuged (9000×g) at 4 °C to obtain CS, and the enterocin was precipitated using 80% (w/v) saturated ammonium sulfate with continuous stirring at 4 °C for 18 h. Precipitated protein pellets were collected by centrifugation (9000×g) and dissolved in sodium acetate buffer (20 mM; pH 4.5). The dissolved precipitates were desalted by passing through Biogel PD-10 column (GE HealthCare, USA), equilibrated and eluted with sodium acetate buffer (20 mM; pH 4.5). The pooled desalted fractions were loaded onto SP-Sepharose Fast Flow cation-exchange column (50× 10 mm; GE Healthcare) and eluted with a linear salt gradient of 0.1 to 1 M NaCl in sodium acetate buffer (20 mM; pH 4.5). The active fractions obtained from SP-Sepharose were pooled, lyophilized, resuspended in HPLC grade water, filtered (0.2 μ pore size) and loaded on the C_18_ HPLC column (Shimadzu Microsorb MV, 100× 10 mm ID, 10 μm). Fractions were eluted with 0.01% trifluoroacetic acid-containing solution of water and acetonitrile in the ratio 30:70 (v/v). The flow rate was maintained at 3 ml/min, and the eluted fractions were monitored by a photodiode array detector at 214 nm. Fractions were collected, concentrated using rotavapour (Buchi, USA) and dissolved in MilliQ water. The antimicrobial activities and protein concentration [57] of the purified fractions were determined after each step of enterocin purification. For all the experiments HPLC fractions were concentrated, pooled and dissolved in appropriate solvent before using.

### Determination of molecular weight and gel overlay assay

To determine the molecular weight of the purified enterocin, denaturing polyacrylamide gel electrophoresis was carried out by using 6% stacking and 15% (w/v) separating gel [58]. The samples and the molecular weight marker (BioRad, USA) were loaded into the wells of the gel. Electrophoresis was carried out at 120 V and lanes of the gel were cut. One lane of the gel was stained with silver nitrate (SRL, India) and the other lane of the gel was used for the overlay assay against *S. enterica* [59]. For the gel overlay assay, BHI soft agar inoculated with *S. enterica* was overlaid onto the lane of the gel, incubated at 37 °C for 24 h, and the zone of inhibition was observed.

### MALDI TOF/TOF mass spectrometry (MS)

For characterization of enterocin, silver-stained gel bands were destained, trypsinised and extracted before subjecting to MALDI-TOF/TOF-Proteomics Analyzer (UltrafleXtreme mass spectrometer; Bruker Daltonics Inc. Germany). Positive ion reflector mode with a mass range from 700 to 3500 Da was used for recording the TOF spectra. For each spectrum 500 shots were accumulated. Two most abundant peptide ions were then subjected to fragmentation analysis. A combined search (MS + LIFT-MS/MS) was performed in database of protein using BioTools 3.0 software (Bruker Daltonics Inc. Germany) and MASCOT (Version 2.1, Matrix Science, London, UK), and searched against all entries in the NCBInr protein databases. The parameters used for the search were as follows: taxonomy, Firmicutes; enzyme, trypsin; the fixed modification, carbamidomethyl (C); the variable modification, Glu->pyro-Glu (N-term Q) and oxidation (M); parent ion mass tolerance at 50 ppm and MS/MS mass tolerance of 0.7 Da; one missed cleavage allowed. The identified proteins among the top hit on the search report with individual ions scores > 44 indicated identity or extensive homology (*p*< 0.05).

### Physico-chemical characterization of purified enterocin 12a

The physico-chemical characterization of purified enterocin 12a (1 μg/ml) dissolved in MilliQ water was carried out. The thermostability of enterocin 12a was determined by incubating it at different temperatures for various time intervals and then determining the residual antimicrobial activity by agar gel diffusion assay. The pH sensitivity of enterocin was determined by setting its pH to values ranging from 2 to 10 and incubating at 37 °C for 1 h. The pH was again set to 6.5 before determining the residual antimicrobial activity. The stability of enterocin 12a to different solvents was determined by incubating it with various organic solvents at 50% v/v for 1 h at ambient temperature. The solvents were removed by vacuum concentration on a rotavapour before determining the residual antimicrobial activity. To study the effect of enzymes on enterocin activity, purified enterocin 12a (1 μg/ml) was treated with different enzymes (proteinase K, pepsin, trypsin, and lipase; Sigma Aldrich, India) at the final concentration of 1 mg/ml for 1 h at 37 °C. The enzymes proteinase K and lipase were dissolved in 100 mM tris-HCL buffer (pH 8.0); whereas for pepsin and trypsin treatment of enterocin, the enzymes were dissolved in 1 mM HCl and enterocin was dissolved in 100 mM tris-HCL buffer (pH 8.0) and the enzyme to protein solution were mixed in the ratio 1:100. After 1 h, enzymes trypsin, pepsin were deactivated by heating at 60 °C for 10 min and the enzymes proteinase k and lipase were inactivated at the temperature of 80 °C for 10 min. The residual antimicrobial activity of enterocin 12a was then determined.

### Effect of enterocin 12a on the cell membrane permeability

Cell permeabilising effects of enterocin 12a on *S. enterica* cells was determined by using flow cytometry with slight modifications [60]. Briefly, *S. enterica* cells suspended in phosphate-buffered saline (PBS; pH 7.2) at a concentration of 1× 10^6^ cells/ml were treated with. Enterocin 12a (5 μg/ml) dissolved in MilliQ water for different time intervals. Following incubation, the cell suspensions were centrifuged, washed and suspended in PBS and thereafter stained with PI (1.0 μg/ml) by incubating for 15 min at 4 °C in the dark. The changes in the fluorescence of the cells was detected by using Flow cytometer (Accuri C6 Flow Cytometer) in the FL2 channel and the data were analyzed using C Flow Plus software (Becton Dickinson, San Jose, CA, USA).

Simultaneously, ten microlitres of PI-stained cell suspension were placed on glass slides in duplicates and fixed with 5 μl Flourmount solution (Sigma). The fixed cells on the glass slides were viewed under a confocal microscope (Nikon, A1R).

### Safety evaluation by hemolytic activity

Hemolytic activity of enterocin 12a dissolved in phosphate- buffered saline (PBS; pH 7.2) was measured spectrophotometrically by hemoglobin release assay [61]. RBCs were isolated from the blood of healthy individuals above the age of 18 yrs. after taking their written informed consent. The protocol was approved by the Institutional Human Ethics Committee of Guru Nanak Dev University, Amritsar, and performed by following the guidelines of the Ethics Committee. Briefly, RBC cell suspension was incubated with different concentrations of enterocin 12a at 37 °C for 1 h. RBC treated with 1% Triton X-100 (Sigma Aldrich), and PBS were used as positive and negative controls, respectively. Hemoglobin release was monitored in the cell supernatant by taking absorbance at 415 nm. The percentage RBC lysis was calculated by using the equation: (A_T_-A_C_)/ (A_X_-A_C_) × 10; Where A_T_ is the absorbance of wells containing enterocin 12a-treated RBC, A_C_ is the absorbance of negative control well having PBS treated-RBC, and A_X_ is the absorbance of positive control well containing 1% Triton X100- treated RBC.

### Assessment of antiproliferative activity of enterocin 12a

MTT assay was used to determine the anti-proliferative activity of enterocin 12a against human cancer cell lines such as HeLa, HCT-15, A549, MG-63, and normal human PBMCs. All the cell lines were procured from National Centre for Cell Science, Pune, India. PBMCs were isolated from the blood of healthy individuals above the age of 18 yrs. after taking their written informed consent. The protocol was approved by the Institutional Human Ethics Committee of Guru Nanak Dev University, Amritsar, India, and performed by following the guidelines of the Ethics Committee. To isolate the PBMCs, density-gradient centrifugation of the blood was done by using Ficoll hypaque [62].

MTT assay was performed according to the protocol described previously [16, 63]. Briefly, cells diluted in Dulbecco’s modified Eagle’s medium (DMEM) containing 10% fetal bovine serum were seeded at the concentration 4× 10^5^ cells/ml in 96-well microtitre plate and incubated for 48 h at 37 °C; 5% CO_2_. Two-fold serial dilutions of purified enterocin 12a dissolved in filter sterlised (0.2 μ pore size) DMEM at concentrations ranging between 5.0 to 0.312 μg/ml were added to the wells and the plate was again incubated for 24 h. The cells were then treated with 100 μl of 0.5 mg/ml MTT (Sigma Aldrich) for 4 h at 37 °C. MTT-containing medium was discarded and 100 μl of dimethyl sulfoxide was added to the wells to dissolve the formazan crystals. The absorbance of the wells was measured at 570 nm on 96-well microplate reader (Synergy™ HT, Bio-Tek Instruments, Inc.). The cells without enterocin were used as controls. The percentage viability of cells was assessed according to the following formula:

$$ \mathrm{Percentage}\ \mathrm{cell}\ \mathrm{viability}=\frac{\mathrm{Absorbance}\ \mathrm{of}\ \mathrm{enterocin}-\mathrm{treated}\ \mathrm{wells}}{\mathrm{Absorbance}\ \mathrm{of}\ \mathrm{untreated}\ \mathrm{control}\ \mathrm{wells}}\times 100 $$

### Apoptosis detection by colorimetric and fluorescent staining

Apoptosis of eukaryotic cells is characterized by morphological changes such as cell shrinkage, chromatin condensation, and nuclear fragmentation [64]. Thus, to study the effect of enterocin 12a on cell morphology, 1.2× 10^6^ HCT-15 cells were cultured on coverslips placed in 6-well tissue culture plate (Costar, USA) at 37 °C; 5% CO_2_. After 24 h, the cells were treated with 1 μg/ml of enterocin 12a and again incubated at 37 °C; 5% CO_2_ for 24 h. After that, the cells were fixed with 4% paraformaldehyde, washed with PBS (pH 7.2) and co-stained with Hoechst 33342 (1 μg/ml; Sigma Aldrich) and PI (5 μg/ml) for 15 min. Separate sets of wells were stained with Giemsa (1:9; Merck, Darmstadt, Germany) for 20 min. The stained cells on the coverslips were viewed under the confocal microscope (Nikon, A1R).

### Statistical analysis

Data in all the experiments are representative of three experiments performed in triplicates. Data were analyzed by independent Student’s *t-*test by using SPSS17.0. Significant differences of means were compared through independent Student’s *t-*test by using SPSS17.0. Individual *p* values for each data set are indicated in each figure.

## Supplementary Information

**Additional file 1 Supplementary Fig. 1**. Silver-stained SDS-PAGE gel showing the bands of partially purified enterocin 12a obtained after SP Sepharose cation-exchange chromatography.

**Additional file 2 Supplementary Fig. 2**. Silver-stained SDS-PAGE gel showing a single band of purified enterocin 12a obtained after reverse phase- HPLC

**Additional file 3 Supplementary Fig. 3**. Agar gel overlay assay of SDS-PAGE gel containing purified enterocin 12a band.

**Additional file 4 Supplementary Table 1**: Antibiotic susceptibility profile of *S. enterica* MTCC 733

**Additional file 5 Supplementary Table 2**. Antibiotic susceptibility profile of *Esc. coli* MTCC119

**Additional file 6 Supplementary Table 3.** Antibiotic susceptibility profile of *Sh. flexneri* MTCC1457

**Additional file 7 Supplementary Table 4:** Antibiotic susceptibility profile of *V. cholerae* MTCC 3906

## Data Availability

All data generated or analysed during this study are included in this published article and its supplementary information files.

## Abbreviations

CS: culture supernatant
MRS: de Man Rogosa and Sharpe medium
RBCs: red blood cells
PBMC: peripheral blood mononuclear cells
PI: propidium iodide
MDR: multi-drug resistant
PBS: phosphate-buffered saline
AU/ml: arbitrary units per ml
RP-HPLC: reverse-phase high-performance liquid chromatography
U-HPLC: ultra-high-performance liquid chromatography

## References

[1] International Agency of Research on Cancer [IARC] (2018). World Cancer report.

[2] Holland T, Fowler VG, Shelburne SA (2014). Invasive gram-positive bacterial infection in cancer patients. Clin Infect Dis.

[3] Alibek K, Bekmurzayeva A, Mussabekova A, Sultankulov B (2012). Using antimicrobial adjuvant therapy in cancer treatment: a review. Infect Agent Cancer.

[4] Rodrigues G, Silva GG, Buccini DF, Duque HM, Dias SC, Franco OL. Bacterial proteinaceous compounds with multiple activities toward cancers and microbial infection. Front Microbiol. 2019;10. 10.3389/fmicb.2019.01690. 10.3389/fmicb.2019.01690 PMC6691048 31447795

[5] Cavera VL, Arthur TD, Kashtanov D, Chikindas ML (2015). Bacteriocins and their position in the next wave of conventional antibiotics. Int J Antimicrob Agents.

[6] Yang SC, Lin CH, Sung CT, Fang JY (2014). Antibacterial activities of bacteriocins: application in foods and pharmaceuticals. Front Microbiol.

[7] Cleveland J, Montville TJ, Nes IF, Chikindas ML (2001). Bacteriocins: safe, natural antimicrobials for 18 h for food preservation. Int J Food Microbiol.

[8] Mathur H, Field D, Rea MC, Cotter PD, Hill C, Ross RP (2017). Bacteriocin-antimicrobial synergy: a medical and food perspective. Front Microbiol.

[9] Hols P, Ledesma-García L, Gabant P, Mignolet J. Mobilization of microbiota commensals and their bacteriocins for therapeutics. Trends Microbiol*.* 2019; doi:org/10.1016/j.tim. 10.1016/j.tim.2019.03.007 30987817

[10] Fernández L, Delgado S, Herrero H, Maldonado A, Rodríguez JM (2008). The bacteriocin nisin, an effective agent for the treatment of *Staphylococcal* mastitis during lactation. J Hum Lact.

[11] Wu J, Hu S, Cao L (2007). Therapeutic effect of nisin Z on subclinical mastitis in lactating cows. Antimicrob Agents Chemother.

[12] Joo NE, Ritchie K, Kamarajan P, Miao D, Kapila YL (2012). Nisin, an apoptogenic bacteriocin and food preservative, attenuates HNSCC tumorigenesis via CHAC1. Cancer Med..

[13] Kaur S, Kaur S (2015). Bacteriocins as potential anticancer agents. Front Pharmacol.

[14] Perez RH, Zendo T, Sonomoto K. Novel bacteriocins from lactic acid bacteria (LAB): various structures and applications. Microb Cell Fact. 2014;13:S3. doi.org/10.1186/1475-2859-13-S1-S3. 10.1186/1475-2859-13-S1-S3 PMC4155820 25186038

[15] Klastersky J (2004). Management of fever in neutropenic patients with different risks of complications. Clin Infect Dis.

[16] Sharma P, Kaur S, Kaur R, Kaur M, Kaur S. Proteinaceous secretory metabolites of probiotic human commensal *Enterococcus hirae* 20c, *E. faecium* 12a and L12b as antiproliferative agents against cancer cell lines. Front Microbiol. 2018;9:948. 10.3389/fmicb.2018.00948 PMC5962654 29867856

[17] Line JE, Svetoch EA, Eruslanov BV, Perelygin VV, Mitsevich EV, Mitsevich IP, Levchuk VP, Svetoch OE, Seal BS, Siragusa GR, Stern NJ (2008). Isolation and purification of enterocin E-760 with broad antimicrobial activity against gram-positive and gram-negative bacteria. Antimicrob Agents Chemother.

[18] Liu H, Zhang L, Yi H, Han X, Gao W, Chi C, Song W, Li H, Liu C (2016). A novel enterocin T1 with anti*-Pseudomonas* activity produced by *Enterococcus faecium* T1 from Chinese Tibet cheese. World J Microbiol Biotechnol.

[19] Casaus P, Nilsen T, Cintas L, Nes I, Hernández P, Holo H (1997). Enterocin B, a new bacteriocin from *Enterococcus faecium* T136 which can act synergistically with enterocin a. Microbiology..

[20] Cintas LM, Casaus P, Håvarstein LS, Hernández PE, Nes IF (1997). Biochemical and genetic characterization of enterocin P, a novel *sec*-dependent bacteriocin from *Enterococcus faecium* P13 with a broad antimicrobial spectrum. Appl Environ Microbiol.

[21] Grande Burgos MJ, Pulido RP, López Aguayo DCM, Gálvez A, Lucas R (2014). The cyclic antibacterial peptide enterocin AS-48: isolation, mode of action, and possible food applications. Int J Mol Sci.

[22] Petri WA, Miller M, Binder HJ, Levine MM, Dillingham R, Guerrant RL (2008). Enteric infections, diarrhea, and their impact on function and development. J Clin Invest.

[23] Magiorakos AP, Srinivasan A, Carey RB, Carmeli Y, Falagas ME, Giske CG, Harbarth S, Hindler JF, Kahlmeter G, Olsson-Liljequist B, Paterson DL (2012). Multidrug-resistant, extensively drug-resistant and pandrug-resistant bacteria: an international expert proposal for interim standard definitions for acquired resistance. Clin Microbiol Infect.

[24] WHO. Antibacterial agents in clinical development: an analysis of the antibacterial clinical development pipeline, including *Mycobacterium tuberculosis*. 2017. WHO/EMP/IAU/2017.11Geneva:WHO.

[25] De Vuyst L, Callewaert R, Crabbé K (1996). Primary metabolite kinetics of bacteriocin biosynthesis by *Lactobacillus amylovorus* and evidence for stimulation of bacteriocin production under unfavourable growth conditions. Microbiology..

[26] Van Tyne D, Martin MJ, Gilmore MS (2013). Structure, function, and biology of the *Enterococcus faecalis* cytolysin. Toxins..

[27] Franz CM, Van Belkum MJ, Holzapfel WH, Abriouel H, Gálvez A (2007). Diversity of enterococcal bacteriocins and their grouping in a new classification scheme. FEMS Microbiol Rev.

[28] Nilsen T, Nes IF, Holo H (2003). Enterolysin a, a cell wall-degrading bacteriocin from *Enterococcus faecalis* LMG 2333. Appl Environ Microbiol.

[29] Kurushima J, Nakane D, Nishizaka T, Tomita H (2015). Bacteriocin protein Bacl1 of *Enterococcus faecalis* targets cell division loci and specifically recognizes L-Ala2-cross-bridged peptidoglycan. J Bacteriol.

[30] Phumisantiphong U, Siripanichgon K, Reamtong O, Diraphat P (2017). A novel bacteriocin from *Enterococcus faecalis* 478 exhibits a potent activity against vancomycin-resistant enterococci. PLoS One.

[31] Shekh RM, Roy U (2012). Biochemical characterization of an anti-*Candida* factor produced by *Enterococcus faecalis*. BMC Microbiol.

[32] Vasilchenko AS, Vasilchenko AV, Valyshev AV, Rogozhin EA. A novel high-molecular-mass bacteriocin produced by *Enterococcus faecium*: biochemical features and mode of action. Probiotics Antimicrob Proteins. 2018:1–8. 10.1007/s12602-018-9392-0 29423898

[33] Lill R (2009). Function and biogenesis of iron-Sulphur proteins. Nature..

[34] Grinter R, Milner J, Walker D (2012). Ferredoxin containing bacteriocins suggest a novel mechanism of iron uptake in *Pectobacterium* spp. PLoS One.

[35] Grinter R, Josts I, Zeth K, Roszak AW, McCaughey LC, Cogdell RJ, Milner JJ, Kelly SM, Byron O, Walker D (2014). Structure of the atypical bacteriocin pectocin M2 implies a novel mechanism of protein uptake. Mol Microbiol.

[36] Ghequire MG, Kemland L, Anoz-Carbonell E, Buchanan SK, De Mot R (2017). A natural chimeric *Pseudomonas* bacteriocin with novel pore-forming activity parasitizes the ferrichrome transporter. MBio..

[37] de Been M, van Schaik W, Cheng L, Corander J, Willems RJ (2013). Recent recombination events in the core genome are associated with adaptive evolution in *Enterococcus faecium*. Genome Biol Evol.

[38] Keren T, Yarmus M, Halevy G, Shapira R (2004). Immunodetection of the bacteriocin lacticin RM: analysis of the influence of temperature and tween 80 on its expression and activity. Appl Environ Microbiol.

[39] Weeks ME, von Caron GN, James DC, Smales CM, Robinson GK (2006). Monitoring changes in nisin susceptibility of *Listeria monocytogenes* Scott a as an indicator of growth phase using FACS. J Microbiol Methods.

[40] Dalmau M, Maier E, Mulet N, Vinas M, Benz R (2002). Bacterial membrane injuries induced by lactacin F and nisin. Int Microbiol.

[41] Ankaiah D, Esakkiraj P, Perumal V, Ayyanna R, Venkatesan A (2017). Probiotic characterization of *Enterococcus faecium* por1: cloning, over expression of Enterocin-a and evaluation of antibacterial, anticancer properties. J Funct Foods.

[42] Villarante KI, Elegado FB, Iwatani S, Zendo T, Sonomoto K, de Guzman EE (2011). Purification and characterization and *in vitro* cytotoxicity of the bacteriocin from *Pediococcus acidilactici* K2a2-3 against human colon adenocarcinoma (HT29) and human cervical carcinoma (HeLa) cells. World J Microbiol Biotechnol.

[43] Sand SL, Nissen-Meyer J, Sand O, Haug TM (1828). Plantaricin a, a cationic peptide produced by *Lactobacillus plantarum*, permeabilizes eukaryotic cell membranes by a mechanism dependent on negative surface charge linked to glycosylated membrane proteins. Biochim Biophys Acta.

[44] Kamarajan P, Hayami T, Matte B, Liu Y, Danciu T, Ramamoorthy A, Worden F, Kapila S, Kapila Y (2015). Nisin ZP, a bacteriocin and food preservative, inhibits head and neck cancer tumorigenesis and prolongs survival. PLoS One.

[45] Joo NE, Ritchie K, Kamarajan P, Miao D, Kapila YI (2012). Nisin, an apoptogenic bacteriocin and food preservative, attenuates HNSCC tumorigenesis via CHAC1. Cancer Med.

[46] Yamada T, Goto M, Punj V, Zaborina O, Kimbara K, Gupta TD, Chakrabarty AM (2002). The bacterial redox protein azurin induces apoptosis in J774 macrophages through complex formation and stabilization of the tumor suppressor protein p53. Infect Immun.

[47] Yang DS, Miao XD, Ye ZM, Feng J, Xu RZ, Huang X, Ge FF (2005). Bacterial redox protein azurin induce apoptosis in human osteosarcoma U2OS cells. Pharmacol Res.

[48] Chakrabarty AM (2016). Bacterial azurin in potential cancer therapy. Cell Cycle.

[49] Dobrzyńska I, Szachowicz-Petelska B, Figaszewski Z, Sulkowski S (2005). Changes in electric charge and phospholipid composition in human colorectal cancer cells. Mol Cell Biochem.

[50] Hoskin DW, Ramamoorthy A (2008). Studies on anticancer activities of antimicrobial peptides. Biochem Biophys Acta.

[51] Sok M, Sentjurc M, Schara M (1999). Membrane fluidity characteristics of human lung cancer. Cancer Lett.

[52] Chaudhary J, Munshi M (1995). Scanning electron microscopic analysis of breast aspirates. Cytopathology..

[53] Leite M, Quinta-Costa M, Leite PS, Guimarães JE (1999). Critical evaluation of techniques to detect and measure cell death-study in a model of UV radiation of the leukaemic cell line HL60. Anal Cell Pathol.

[54] Punj V, Bhattacharyya S, Saint-Dic D, Vasu C, Cunningham EA, Graves J, Yamada T, Constantinou AI, Christov K, White B, Li G (2004). Bacterial cupredoxin azurin as an inducer of apoptosis and regression in human breast cancer. Oncogene..

[55] Kommineni S, Bretl DJ, Lam V, Chakraborty R, Hayward M, Simpson P, Cao Y, Bousounis P, Kristich CJ, Salzman NH (2015). Bacteriocin production augments niche competition by enterococci in the mammalian gastrointestinal tract. Nature..

[56] Geis A, Singh J, Teuber M (1983). Potential of lactic streptococci to produce bacteriocin. Appl Environ Microbiol.

[57] Bradford MM (1976). A rapid and sensitive method for the quantitation of microgram quantities of protein utilizing the principle of protein-dye binding. Anal Biochem.

[58] Laemmli UK (1970). Cleavage of structural proteins during the assembly of the head of bacteriophage T4. Nature..

[59] Dezwaan DC, Mequio MJ, Littell JS, Allen JP, Rossbach S, Pybus V (2007). Purification and characterization of enterocin 62-6, a two-peptide bacteriocin produced by a vaginal strain of *Enterococcus faecium*: potential significance in bacterial vaginosis. Microb Ecol Health Dis.

[60] Chopra L, Singh G, Jena KK, Sahoo DK (2015). Sonorensin: a new bacteriocin with potential of an anti-biofilm agent and a food biopreservative. Sci Rep.

[61] Paiva AD, de Oliveira MD, de Paula SO, Baracat-Pereira MC, Breukink E, Mantovani HC (2012). Toxicity of bovicin HC5 against mammalian cell lines and the role of cholesterol in bacteriocin activity. Microbiology..

[62] Hessle C, Andersson B, Wold AE (2000). Gram-positive bacteria are potent inducers of monocytic interleukin-12 (IL-12) while gram-negative bacteria preferentially stimulate IL-10 production. Infect Immun.

[63] Mosmann T (1983). Rapid colorimetric assay for cellular growth and survival: application to proliferation and cytotoxicity assays. J Immunol Methods.

[64] Elmore S (2007). Apoptosis: a review of programmed cell death. Toxicol Pathol.

