# Supplementary material for: Anticancer and antimicrobial potential of enterocin 12a from Enterococcus faecium
Source: BMC Microbiol. 2021 Feb 4;21:39. doi: 10.1186/s12866-021-02086-5 (PMC7860584; doi:10.1186/s12866-021-02086-5)
Supplement: Supplementary file 1 — Additional file 1 Supplementary Fig. 1. Silver-stained SDS-PAGE gel showing the bands of partially purified enterocin 12a obtained after SP Sepharose cation-exchange chromatography. [file 12866_2021_2086_MOESM1_ESM.docx]

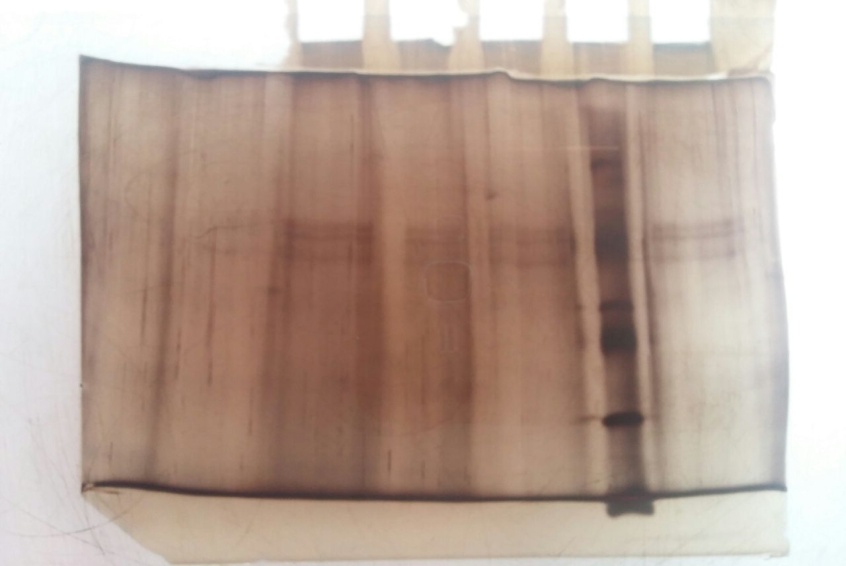


97.4 kDa

31 kDa

45 kDa

66.2 kDa

Visible bands

21.5 kDa

**Supplementary Fig 1:** Image of the silver-stained SDS-PAGE gel showing the bands of partially purified enterocin 12a obtained after SP Sepharose cation-exchange chromatography. **Lanes 1-3** show bands of cation-exchange fractions eluted with 0.4 mM NaCl containing sodium acetate buffer. **Lane 4** shows the molecular weight marker.
