# Supplementary material for: Anticancer and antimicrobial potential of enterocin 12a from Enterococcus faecium
Source: BMC Microbiol. 2021 Feb 4;21:39. doi: 10.1186/s12866-021-02086-5 (PMC7860584; doi:10.1186/s12866-021-02086-5)
Supplement: Supplementary file 2 — Additional file 2 Supplementary Fig. 2. Silver-stained SDS-PAGE gel showing a single band of purified enterocin 12a obtained after reverse phase- HPLC [file 12866_2021_2086_MOESM2_ESM.docx]

**
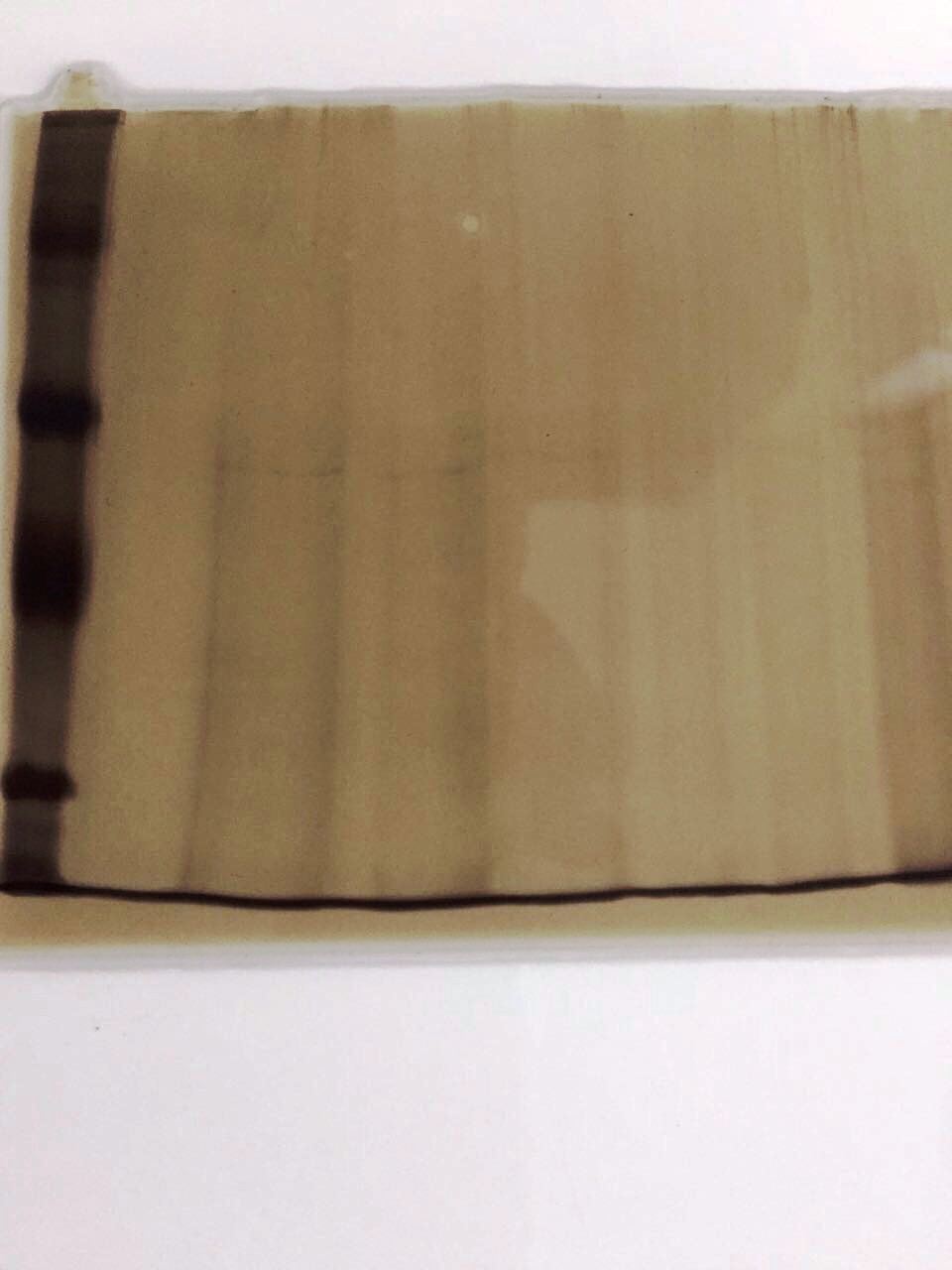
**

45 kDa

21.5 kDa

31 kDa

66.2 kDa

Enterocin 12a band

Lane 1

Lane 2

97.4 kDa

**Supplementary Fig** **2:** Image of the silver-stained SDS-PAGE gel showing a single band of purified enterocin 12a obtained after reverse phase- HPLC. **Lane 1** shows the molecular weight marker. **Lane 2** shows the band obtained after RP-HPLC.
