# Supplementary material for: Anticancer and antimicrobial potential of enterocin 12a from Enterococcus faecium
Source: BMC Microbiol. 2021 Feb 4;21:39. doi: 10.1186/s12866-021-02086-5 (PMC7860584; doi:10.1186/s12866-021-02086-5)
Supplement: Supplementary file 3 — Additional file 3 Supplementary Fig. 3. Agar gel overlay assay of SDS-PAGE gel containing purified enterocin 12a band. [file 12866_2021_2086_MOESM3_ESM.docx]

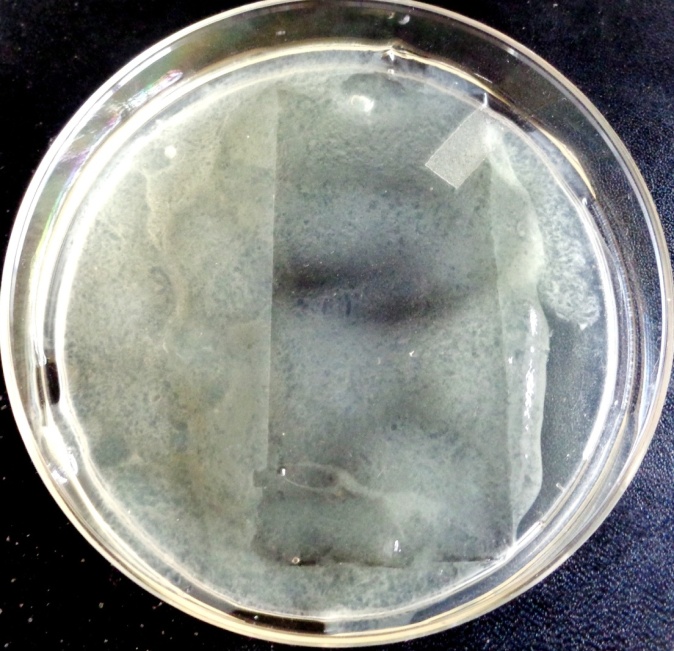


**Supplementary Fig** **3**: Agar gel overlay assay of SDS-PAGE gel containing purified enterocin 12a band. The image shows the zone of inhibition around the purified enterocin 12a band*.* *S. enterica* was used as the indicator culture.
