# Supplementary material for: Anticancer and antimicrobial potential of enterocin 12a from Enterococcus faecium
Source: BMC Microbiol. 2021 Feb 4;21:39. doi: 10.1186/s12866-021-02086-5 (PMC7860584; doi:10.1186/s12866-021-02086-5)
Supplement: Supplementary file 4 — Additional file 4 Supplementary Table 1: Antibiotic susceptibility profile of S. enterica MTCC 733 [file 12866_2021_2086_MOESM4_ESM.docx]

**Supplementary Table 1:** Antibiotic susceptibility profile of *S. enterica* MTCC 733

| **Antibiotic** | **Concentration (µg/ml)** | **Zone of inhibition(mm)** | **Susceptibility profile** |
| --- | --- | --- | --- |
| **ß-lactams** |  |  |  |
| Penicillin | 2U | 17±0.1 | R |
| Ampicillin | 10 | 33±0.09 | S |
| Methicillin | 5 | -* | R |
| **Fluoroquinolones** |  |  |  |
| Ofloxacin | 5 | 7±0.12 | R |
| Ciprofloxacin | 5 | 16±0.71 | I |
| Norfloxacin | 10 | 20±0.06 | S |
| Gatifloxacin | 5 | 18±0.11 | S |
| Sparfloxacin | 5 | - | R |
| Moxifloxacin | 5 | 17±0.32 | R |
| **Macrolides** |  |  |  |
| Azithromycin | 15 | - | R |
| Clarithromycin | 15 | - | R |
| **Aminoglycosides** |  |  |  |
| Kanamycin | 30 | 9±0.02 | R |
| Streptomycin | 10 | - | R |
| Gentamicin | 120 | 20±0.89 | R |
| Amikacin | 30 | 22±0.61 | S |
| **Cephalosporins** |  |  |  |
| Cefexime | 5 | - | R |
| **Oxazolidinones** |  |  |  |
| Linezolid | 15 | 46±0.52 | S |
| **Others** |  |  |  |
| Colistin | 10 | - | R |
| Tetracycline | 30 | 20±0.2 | S |

-* no zone of inhibition observed

Kirby Bauer disc diffusion method was performed, and zones of inhibition were measured in mm. The standard interpretation of antimicrobial susceptibility tests was done by following EUCAST standards (2018). **S:** Susceptible; **I:** Intermediate; **R:** Resistant.
